# Multi-scale modelling to evaluate building energy consumption at the neighbourhood scale

**DOI:** 10.1371/journal.pone.0183437

**Published:** 2017-09-07

**Authors:** Dasaraden Mauree, Silvia Coccolo, Jérôme Kaempf, Jean-Louis Scartezzini

**Affiliations:** 1 Solar Energy and Building Physics Laboratory. Ecole Polytechnique Fédérale de Lausanne, CH-1015 Lausanne, Switzerland; 2 Haute Ecole d’Ingénierie et d’Architecture, HES-SO Fribourg, Bd de Perolles 80, 1705 Fribourg, Switzerland; Centro de Investigacion Cientifica y de Educacion Superior de Ensenada Division de Fisica Aplicada, MEXICO

## Abstract

A new methodology is proposed to couple a meteorological model with a building energy use model. The aim of such a coupling is to improve the boundary conditions of both models with no significant increase in computational time. In the present case, the Canopy Interface Model (CIM) is coupled with CitySim. CitySim provides the geometrical characteristics to CIM, which then calculates a high resolution profile of the meteorological variables. These are in turn used by CitySim to calculate the energy flows in an urban district. We have conducted a series of experiments on the EPFL campus in Lausanne, Switzerland, to show the effectiveness of the coupling strategy. First, measured data from the campus for the year 2015 are used to force CIM and to evaluate its aptitude to reproduce high resolution vertical profiles. Second, we compare the use of local climatic data and data from a meteorological station located outside the urban area, in an evaluation of energy use. In both experiments, we demonstrate the importance of using in building energy software, meteorological variables that account for the urban microclimate. Furthermore, we also show that some building and urban forms are more sensitive to the local environment.

## Introduction

Cities have a crucial role to play in the mitigation and adaptation to climate change with the development of more sustainable urban planning scenarios. It is now well known that building energy demand and urban climate are closely related and interdependent [1, 2]. The urban climate depends on a series of processes taking place at different spatial (from global to local) and temporal scales [3]. Due to this complexity, models have been developed in recent years to better understand and simulate these processes.

Meteorological mesoscale models were initially dedicated to weather forecasting without the need to detail interactions between urban areas and the atmosphere [4]. In the last few years, urban parametrizations have been integrated in these mesoscale models to also simulate urban heat islands (UHI) [5–7], building energy consumption [2, 8] and air pollution at the urban scale [4]. The underlying purpose is to develop systems that help urban planners make informed decisions and propose sustainable urban planning scenarios to decrease UHIs, building energy demand, or urban air pollution. However, these models often lack a proper representation of the surface and obstacles and thus do not consider the appropriate surface temperature in urban areas.

The development and use of microscale models, working at the building or neighbourhood scale, (such as Envimet [9], EnergyPlus [10] or CitySim [11, 12] for the evaluation of energy use at a preliminary stage in the design process have considerably increased. Such models are commonly forced with climatic conditions averaged over several years and collected outside of urban areas. Consequently, they do not take into account the local climatic conditions prevailing on site, and the exchanges between buildings and the outdoor environment are not evaluated properly.

In order to overcome this problem, multiscale modelling could be the solution. In previous studies [13, 14], a new one-dimensional Canopy Interface Model (CIM) was developed and it was shown that it could produce a high-resolution vertical profile for wind, potential temperature and humidity. Furthermore, it was integrated in the meteorological mesoscale model WRF [15] to improve the surface representation in such models [16].

In the present study, CIM is coupled to CitySim to improve the climatic boundary conditions used in the calculation of the energy balance. CIM will provide high-resolution vertical meteorological profiles to CitySim while CitySim will provide a better representation of the surface and building characteristics. In the next section, we give a brief description of the different models and the coupling methodology. We then present the experiments that we conducted to validate our coupling and how we used the proposed system to evaluate planning scenarios. Finally, we conclude and give some perspectives for this work.

## Materials and methods

In this section, we give a brief description of the models used in the current study and the methodology used to couple them.

### CitySim

#### Brief description

CitySim is an urban energy modelling tool [17], able to quantify the energy demand from the building to the city scale. The thermal model of buildings is based on an analogy with the electrical circuit, or more precisely on a simplified resistor-capacitor network [11, 12]. The radiation model, previously validated with Radiance, is based on the Simplified Radiosity Algorithm (SRA). With the SRA, the radiant external environment is represented by two hemispheres, discretized into several solid angles [18]. CitySim provides the energy needs of buildings, as well as the electricity demand and the energy produced by renewable energy sources. Results obtained by the software were previously validated with the BESTEST, showing a sound correlation between them [19]. CitySim works dynamically, providing the results in hourly values, and by including the interactions within the built environment. Among other, the inter-reflections between buildings’ surfaces as well as the mutual shading are calculated. In order to perform the calculations, hourly weather data are required, such as those generated by the software Meteonorm [20], or by on-site monitoring. Recent development of the model considers the inclusion of the microclimatic conditions, by calculating the evapotranspiration from the ground [21] and the impact of greening on the outdoor human comfort [22].

#### Energy balance

CitySim uses an energy balance of the surface, such as described by Nunez and Oke [23].

$$\begin{array}{c} Q_{sw}+Q_{lw}=Q_{ch}+Q_{et}+Q_{gf} \end{array}$$ (1)

where *Q*_*sw*_, *Q*_*lw*_, *Q*_*ch*_, *Q*_*et*_ and *Q*_*gf*_ are respectively the absorbed shortwave solar radiation by the surface, the longwave radiative flux balance, the convective heat flux from the surface to the air, the evapotranspiration heat flux to the air and the conductive heat flux to the ground. All fluxes are in watts per square meter (W m^−2^). For the purpose of this study, we will be focusing only on the *Q*_*ch*_ term.

#### Heat convection coefficient

A detailed review of the heat convection coefficient can be found in Mirsadeghi et al. [24]. CitySim uses the McAdams formulation for the convective heat transfer coefficient, *h*_*c*_ (W m-2 K-1) and is calculated as follows:

$$\begin{array}{c} h_{c}=2.8+3U \end{array}$$ (2)

where *U* (m s-1) is the wind speed calculated based on the wind attack angle on a particular surface in the windward or leeward-direction [24]. The convective heat flux, *Q*_*ch*_, is then calculated as:

$$\begin{array}{c} Q_{ch}=h_{c}\left(T_{s}-T_{air}\right) \end{array}$$ (3)

where *T*_*s*_ (°C) is the surface temperature calculated by CitySim from the energy balance and *T*_*air*_ (°C) is the air temperature as given by the climate file.

### Canopy Interface Model

The Canopy Interface Model (CIM) is a 1D-column module that was developed by Mauree et al. [14].

#### Governing equations

CIM calculates values for the wind speed and air temperature along the vertical using differential equations for the momentum and the potential temperature:

$$\begin{array}{c} \frac{du}{dt}=\frac{d}{dz}(\mu_{t}\frac{du}{dz})+f_{u}^{s} \end{array}$$ (4)

$$\begin{array}{c} \frac{d\theta }{dt}=\frac{d}{dz}(\kappa_{t}\frac{d\theta }{dz})+f_{\theta }^{s}, \end{array}$$ (5)

where *u* is the mean horizontal velocity (m s^−1^), *θ* is the potential temperature (K), *μ*_*t*_ and *κ*_*t*_ are the momentum and heat viscosity coefficients (calculated using a 1.5 turbulence closure) and $f_{u}^{s}$ and $f_{\theta }^{s}$ are the source terms representing the fluxes that will impact the flow.

#### Heat convection coefficient

The heat fluxes from vertical walls are function of the wind speed and the difference between the air and surface temperature. In the present study, we adopt the same formulation as used by CitySim in order to be coherent with its calculation of the convection heat transfer coefficient. The formulation, for the convective heat flux, $F\theta_{I}^{{vert}_{x}}$, is given here only for the *x*-direction.

$$\begin{array}{c} F\theta_{I}^{{vert}_{x}}=-\left(2.8+3\vec{u_{I}}\right)\frac{\Delta \theta_{I}^{V}}{C_{p}}\frac{{\hat{\varphi }}_{{vert}_{x}}}{\phi }, \end{array}$$ (6)

where $\vec{u_{I}}$ is the horizontal wind vector at the centre of the *I^th^* level, $\Delta \theta_{I}^{V}$ is the difference between the air temperature and the temperature of the vertical surface at the level *I* in the *x*-direction, *C*_*p*_ is the air heat capacity and is taken as 1004 J kg^−1^ K^−1^, $\;{\hat{\varphi }}_{{vert}_{x}}$ is the surface perpendicular to the wind direction and *ϕ* the free volume porosity at each level. Please refer to Mauree et al. [14] for a more detailed description of the additional forces impacting the flow as well as the mixing length.

### Coupling strategy

**Fig 1:**
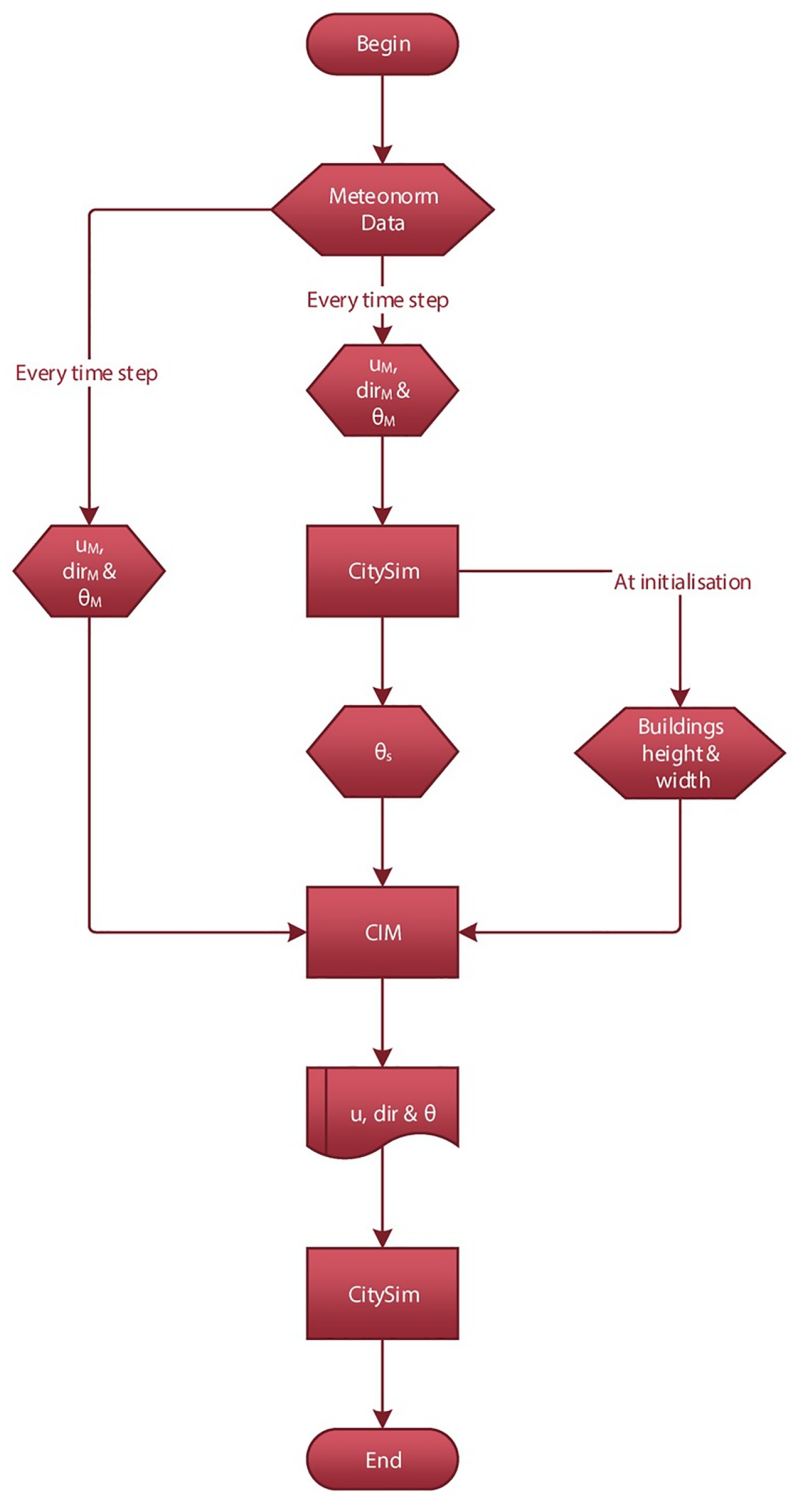
Flowchart describing the coupling strategy CIM-CitySim. *u*_*M*_ is the horizontal wind speed, *dir*_*M*_ is the wind direction and *θ*_*M*_ the air temperature as provided by Meteonorm, *θ*_*s*_ is the surface temperature and *u* is the wind speed, *dir* the wind direction and *θ* the air temperature as calculated by CIM.

In this study a coupling between CIM and CitySim is performed. At initialization, CitySim provides geometrical information about the considered study case. For every time step, CitySim calculates the surface temperature to CIM using the meteorological values from Meteonorm. CIM then uses these surface temperatures as well as the wind speed, direction and air temperature as boundary conditions to compute high resolution vertical profiles. The meteorological variables computed by CIM are then used as input for CitySim, which performs its final simulation of the energy consumption. Fig 1 illustrates the strategy adopted for this study.

## Experiments

### Validation of CIM with meteorological data

In order to validate the above methodology, a 1D column for CIM is setup with 20 cells of 3m each along the vertical. CIM is used for the current study in an offline mode and is forced at the top most cell. A traditional log law is used to obtain values at 62.5m (corresponding to the height of the top most cell of CIM). Simulations are run for the year 2015 and the surface temperatures are obtained from a CitySim simulation. Two meteorological stations located on the EPFL campus near the LESO-PB buildings have provided the values for wind speed, direction and air temperature at 2m and 12m for the year 2015. The calculation from CIM are then compared with experimental data.

### Energy modelling of the EPFL campus: Setup and validation

**Fig 2:**
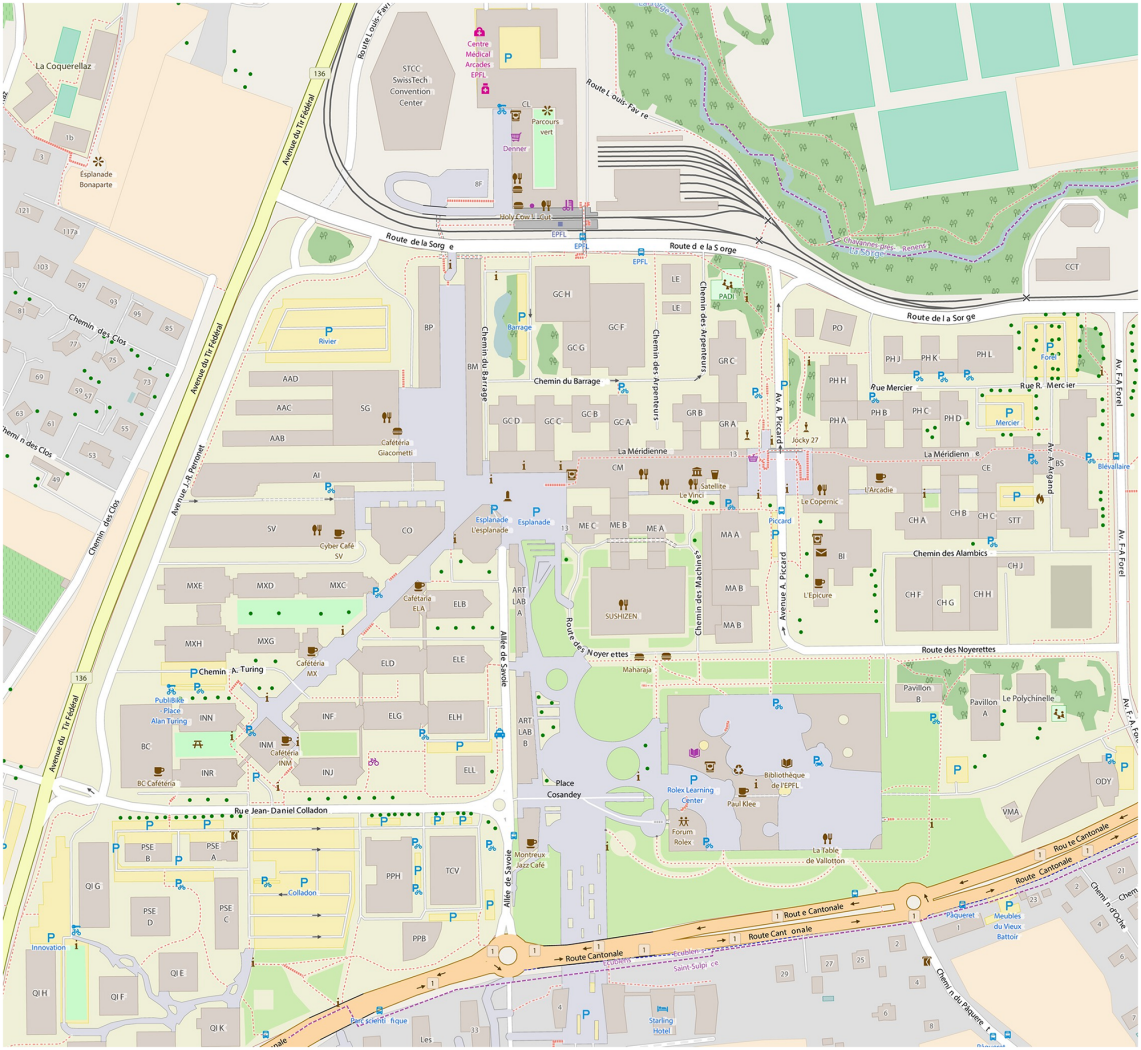
Map of the EPFL campus, Switzerland (Extracted from plan.epfl.ch). This image is taken from Open Street Map whose copyright notices can be found here: https://www.openstreetmap.org/copyright (CC-BY-SA-2.0).

In order to understand the impact of correct climatic data on energy simulations, analyses are made for the existing EPFL campus, in Lausanne, Switzerland (see Fig 2) to quantify the sensitivity of the built stock to the wind profile as well as to generalize the results as a function of the envelopes. The objective is to find which buildings are more sensitive to the local wind and temperature variations. Two scenarios are run: (i) using the Meteonorm data and (ii) using the CIM-CitySim data.

We present here the setup of the model, as well as the physical characteristics of the campus. The energy model of the campus was set up in a previous study [25]: hourly heating and cooling demand of the site were provided by the software CitySim, and validated with on-site monitoring for the years 2011, 2012 and 2013. The geometrical information of the campus was retrieved from Carneiro [26], and the physical data of the buildings were defined according to the phase of construction. The campus was built in three main phases, and each phase of construction corresponds to a homogenous architectural plan. We hence applied to each building the physical characteristics that correspond to its period of construction. The envelopes of each period of construction are defined using the software Lesosai on the basis of imported physical characteristics, as designed in the architectural plan. Examples of buildings characeristics can be found in S1 Table.

### Renovation scenarios

As stated above, the objective of this paper is to understand the impact of local climatic conditions on the energy demand of buildings, as well as its sensitivity as a function of the envelope characteristics. Simulations of a hypothetical refurbishment of the university campus according to the high energy efficiency standard Minergie-P were performed [25]. Minergie [27] is a well-established standard, commonly applied to the Swiss construction market; a further improvement of this standard is the so called Minergie-P, which implies a lower energy demand. In order to apply the standard, all buildings are well insulated with 35cm of EPS and triple glazing with infrared coating. The novelty in the proposed approach is the fact that the Minergie standard is applied to an entire campus, not only to one building. Based on the simulation results, the impact of refurbishment on the energy consumption and its sensitivity to the local climatic data is then analysed.

### Urban form

To further understand the impact of local meteorological variables on the energy demand, five urban configurations are selected (see Table 1), adapted from [28]; the district is included in a square of 100 m per each side. Their urban characteristics, taken from [29], are the following: i) ground floor area (m2), ii) number of floors (-), iii) treated floor area (m2), iv) Floor Area Ratio, or Plot Ratio (-), v) Site Coverage (%) and vi) Form Factor (-). The Floor Area Ratio (FAR), or Plot Ratio, is defined as ratio of the gross floor area to the site area, the Site Coverage (SC) is defined as the ratio of buildings footprint to the site area [30] and the Form Factor is defined as follows [31]:

$$\begin{array}{c} FF=\frac{W+R+0.5G}{A} \end{array}$$ (7)

where *W* is the wall area, *R* is the roof area, *G* is the ground area and *A* is the gross area of the building. All values are expressed in m^2^.

**Table 1:** Urban characteristics of each case study from [28]. (G—Ground floor area, N—Number of floors, TFA—Treated floor area, FAR—Floor area ratio, SC—Site coverage, FF—Form factor).

Case Study	G (m^2^)	N (-)	TFA (m^2^)	FAR (-)	SC (%)	FF (-)
A	900	4	3600	0.036	0.009	1.37
B	3000	4	12000	0.12	0.03	0.95
C	1500	4	6000	0.06	0.015	1.02
D	4050	4	16200	0.162	0.04	0.87
E	3000	4	12000	0.12	0.03	0.87
F	5100	4	20400	0.20	0.05	0.87

The selected configurations are then simulated with CitySim Pro, in order to quantify their energy demand, by varying the outdoor air temperature as well as the wind speed.

## Results and discussions

### CIM validation with data from LESO

CIM is run for one full year (2015) and the boundary conditions are used for the top-most cell of the 1D column of CIM and for the surface temperature of the obstacles. The results are then compared at two points (2 m and 12 m) along a vertical axis for which the measurements were available.

**Fig 3:**
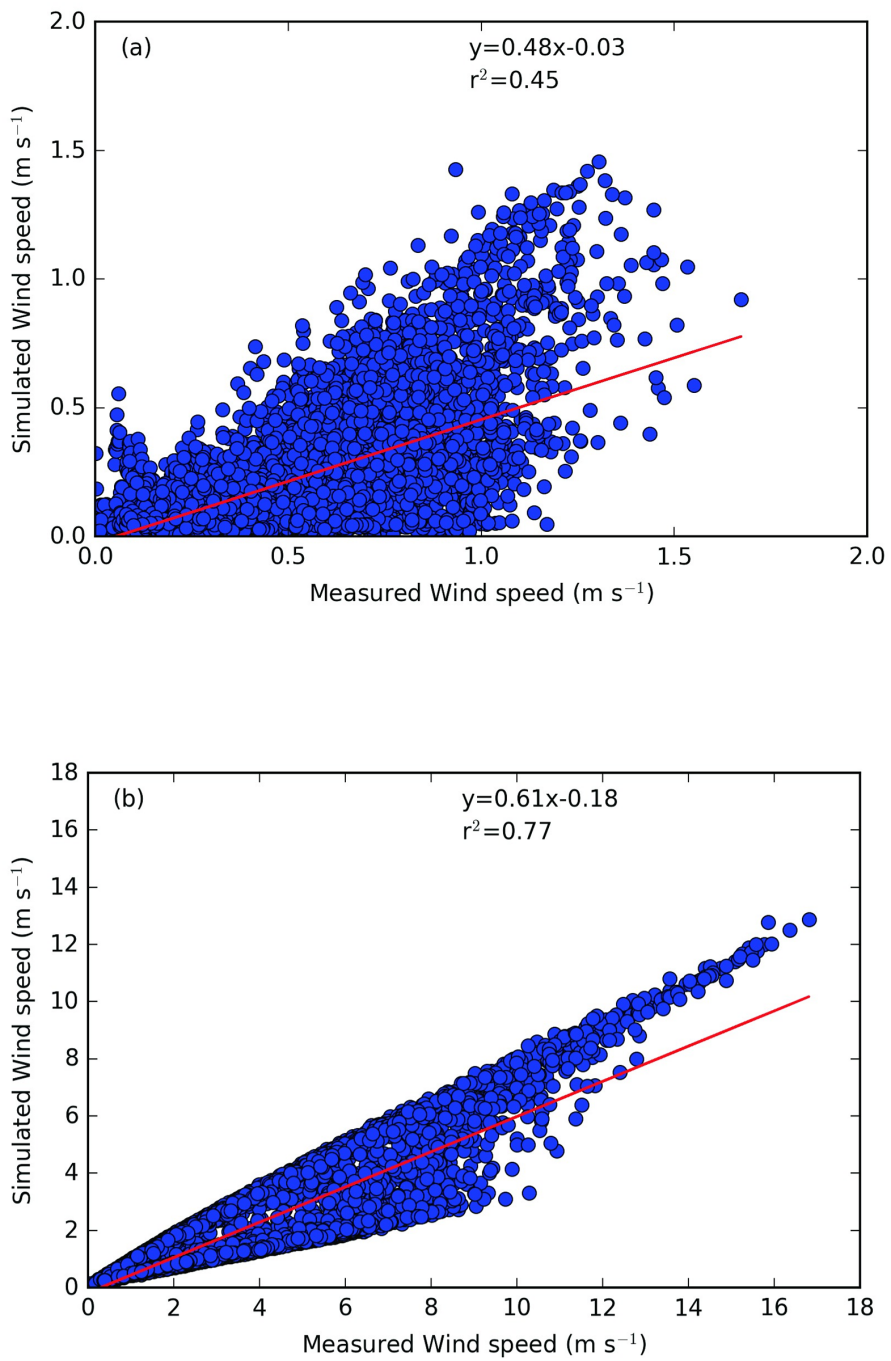
Comparison of measured and simulated wind speed (in m s^−1^) for the year 2015 at (a) 2m and (b) 12m above ground.

**Fig 4:**
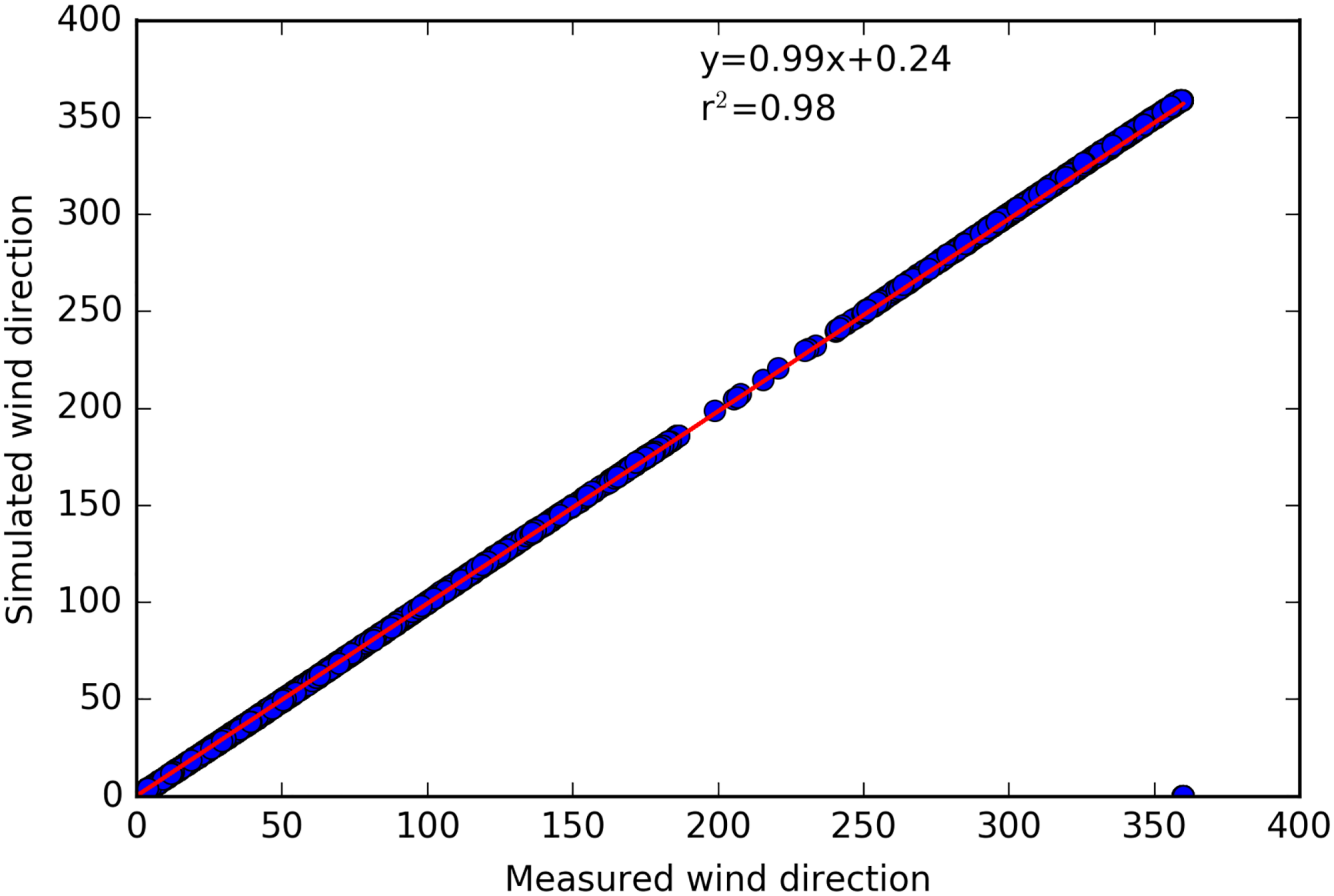
Comparison of measured and simulated wind direction (in °) for the year 2015 at 12m above ground.

**Fig 5:**
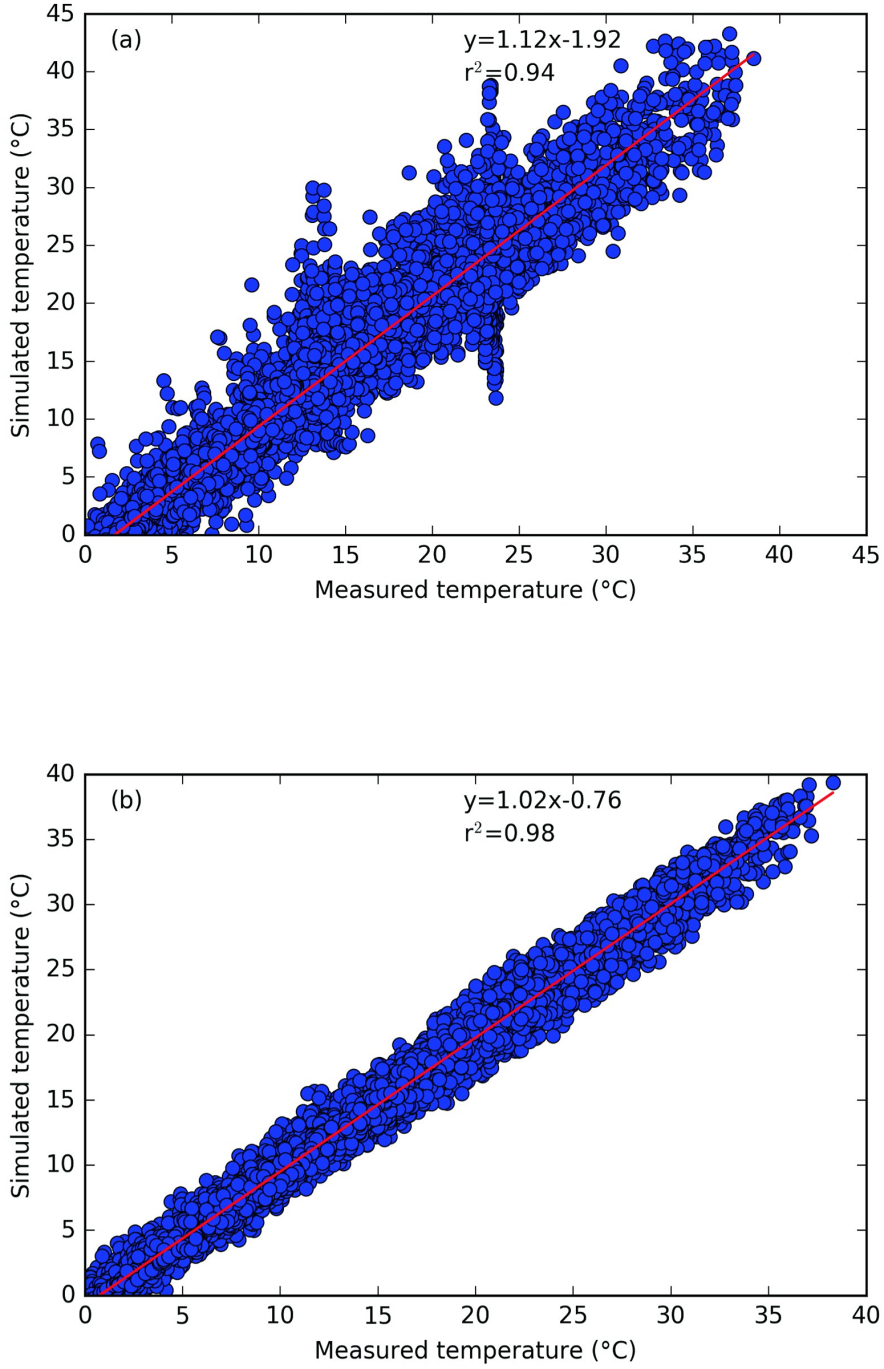
Comparison of measured and simulated temperature (in °C) for the year 2015 at (a) 2m and (b) 12m above ground.

Figs 3, 4 and 5 show the horizontal wind speed, the wind direction and the air temperature, respectively, calculated from CIM as well as the measured data from two positions. It can be seen that overall there is a very good correspondence between the simulated and the measured data. Note that due to the high turbulence and variability of the wind close to the ground, there was no coherence between the measured and simulated wind direction at 2m. CIM only resolves the flow in a 1D column and hence in two horizontal directions. It hence does not capture the complex 3 dimensional behaviour of the wind close to the surface and in between buildings but can simulate with limited computational resources the general patterns. Table 2 gives the correlation, bias and root mean squarred errror for the wind speed and temperature at 2 m and 12 m and the wind direction at 12m between the measured and simulated value. It can clearly be seen from these values that CIM can calculate with a high accuracy the wind and temperature profiles with limited information regarding the geometrical characteristics given as input data. Furthermore, these results are a good indication of the effective coupling between CIM and CitySim. The surface temperatures calculated by CitySim are providing valuable data to CIM in order to create a dynamic simulation that is also useful to determine the annual and seasonal variation.

**Table 2:** Correlation, bias and RMSE (root mean square error) between measured and simulated data from CIM.

	Wind speed		Wind direction	Air Temperature	
	2m	12m	12m	2m	12m
Correlation	0.67	0.88	0.99	0.97	0.99
r^2^	0.45	0.77	0.98	0.94	0.98
Bias	0.27	1.46	0.71	0.34	0.42
RMSE	0.34	1.96	14.3	2.71	1.22

While CIM is able to reproduce the general trend of the wind speed (bias of 0.27 at 2m and 1.46 at 12 m), there is an underestimation of the wind speed at these heights above ground. One of the reasons for this might be the inherent capacity of CIM to calculate horizontally averaged profiles, while we here have point measurements on top of a building, which are more likely to capture more turbulent effects (such as wake turbulence). Another possible reason could be the calculation of the displacement height. Note also that as CIM is forced at the top, there is a very good correlation for the wind direction above the obstacles. The forces induced by the buildings only influence the magnitude of the wind for each direction and as was also noted by Santiago and Martilli [32], the usual drag force parameterization often leads to a higher than expected flux which then has a stronger impact on the wind speed. However, at 2m, the low wind speed and the high variability along with increased turbulent patterns, makes it difficult for CIM to reproduce perfectly the wind patterns at this height.

### Comparison of CitySim results for energy demand

The energy demand of the EPFL campus is evaluated in two scenarios. First, CitySim calculations are made with data coming from a typical year extracted from the Meteonorm software. Second, the same data is used to force the boundary conditions of CIM to include the urban effects on the meteorological variables and are then used as input for CitySim.

**Fig 6:**
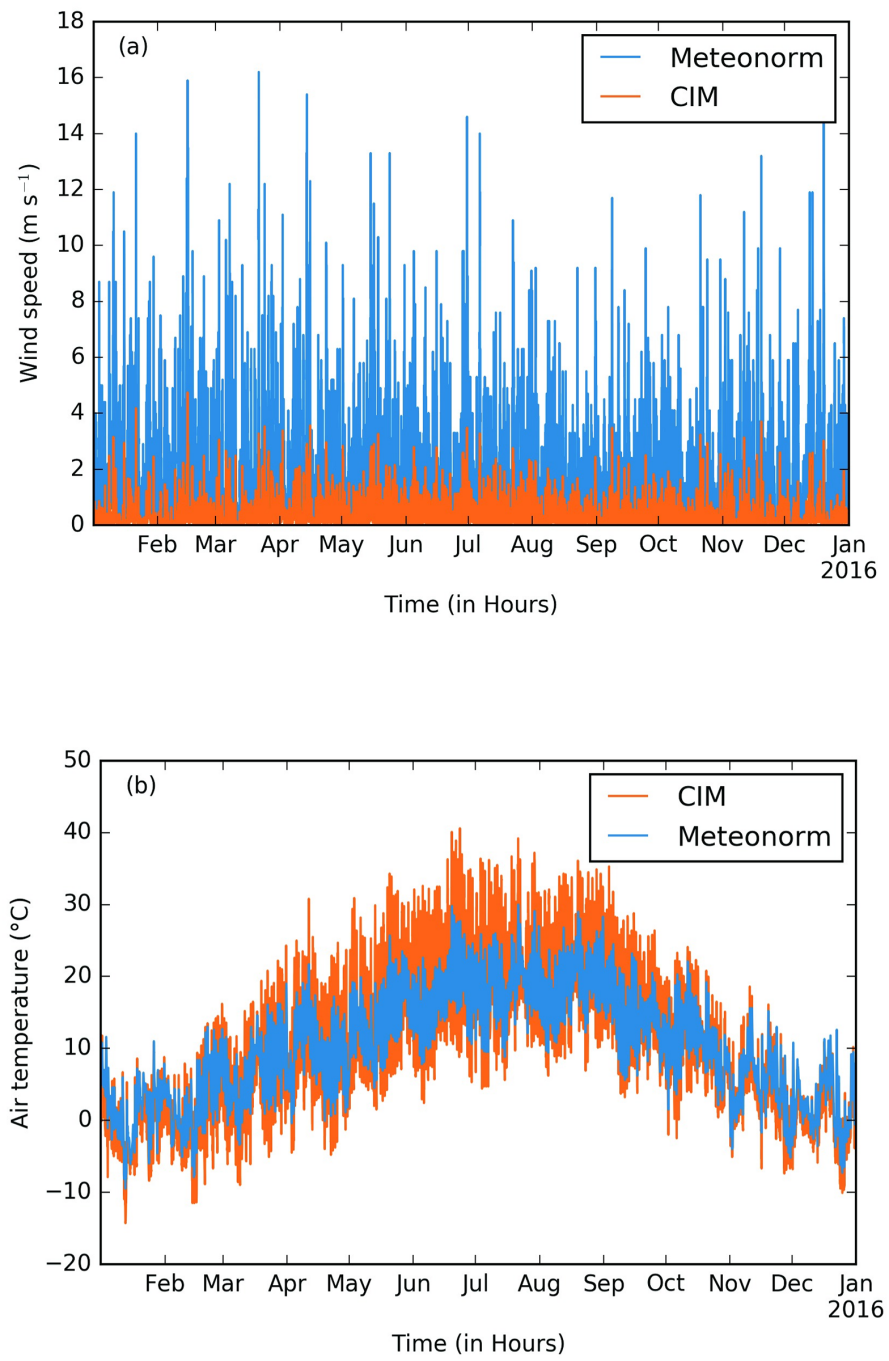
Time series of (a) wind speed (m s^−1^) and (b) the air temperature (°C) used by the CitySim software directly from Meteonorm (in blue) and from CIM (in orange).

Fig 6 shows the two yearly time series for the wind speed and the air temperature used by the CitySim software. It can be seen that the wind speed generated by CIM is usually lower than that generated by Meteonorm. As shown in the previous section, CIM calculates horizontally averaged wind speed and inside the urban canopy there is usually a strong drag force that significantly impacts the wind speed. As for the air temperature it can be noted that although during the winter season there is less difference between the Meteonorm data and the CIM data, during the summer season CIM tends to have a higher air temperature. One reason for this might be the fact that CIM takes into account the surface temperature to calculate its vertical profiles. Due to the lower wind speed, there can be local trapping of heat inside an urban canyon. This would cause an increase in the temperature as is often noted in the urban heat island phenomenon. However, there is also a greater spread of the temperature as CIM captures the daily dynamic of the urban areas.

**Fig 7:**
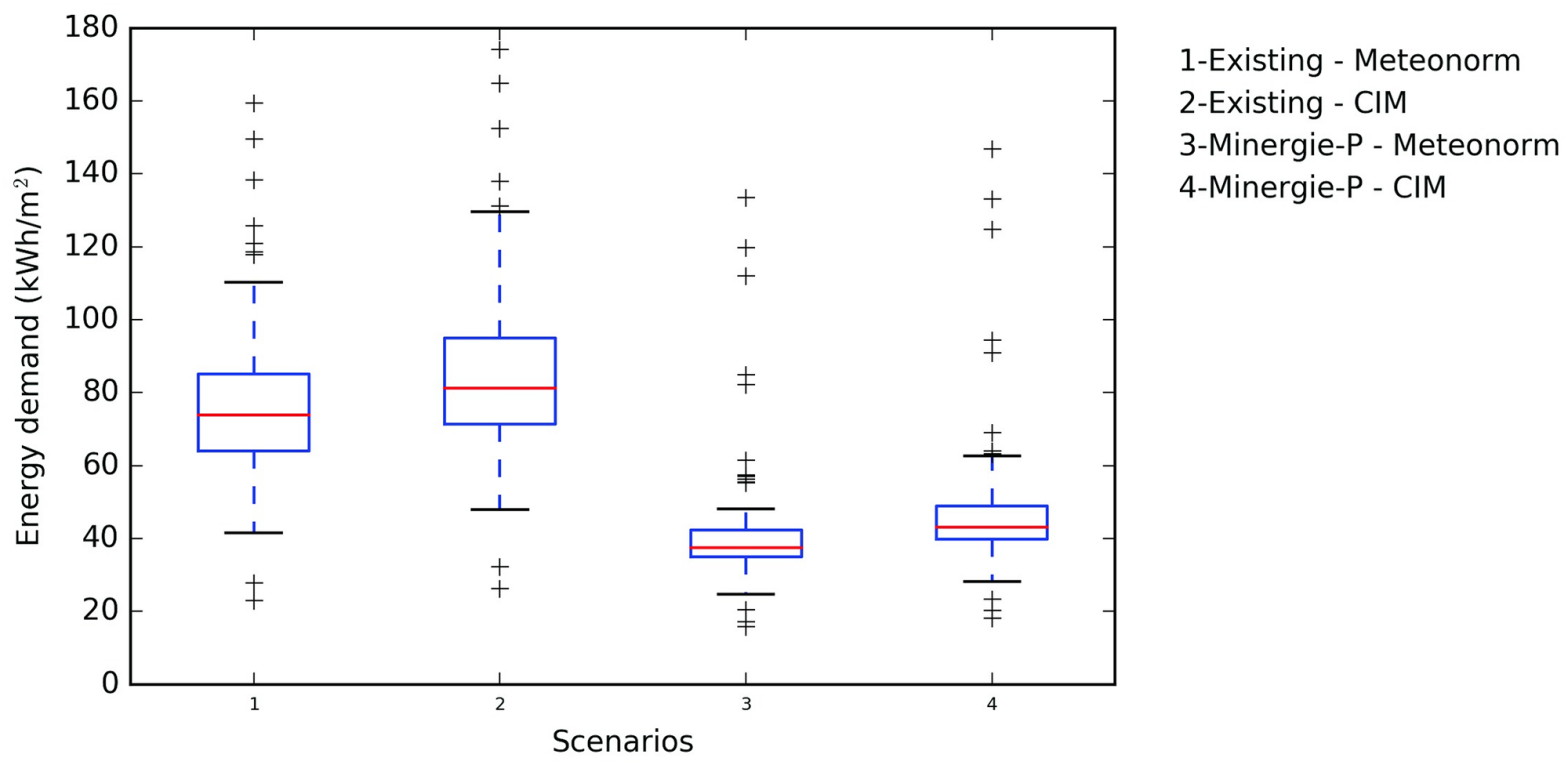
Box plot for the energy demand of EPFL campus. Existing buildings using Meteonorm weather data (1) and CIM weather data (2) and Minergie-P using Meteonorm weather data (3) and CIM weather data (4).

Fig 7 summarizes using box plots (showing the upper and lower quartiles, median values and outliers) the heating demand of buildings with the weather data calculated by Meteonorm and by CIM. The existing campus has an average energy demand for heating equal to 79 kWh m^−2^; assuming the meteorological profiles computed using CIM, the demand increases to 88 kWh m^−2^. The average increase in heating demand for the entire campus corresponds to 9.65%, but the newer buildings are more sensitive to the local meteorological variation: buildings BC and Rolex Learning Centre see their demand increased by 13%. Additionally Fig 7 illustrates that there is a slightly greater spread for the energy demand when using CIM.

The same analyses are conducted for the renovated EPFL campus to meet the Minergie-P standard (see Fig 7); in this case, all buildings are well insulated and equipped with triple glazing with infrared coating. The thermal behaviour of the campus is the same as in the previous case study, showing an increase in the demand by using the CIM weather data. But in this case study, the average demand passes from 44 kWh m^−2^ to 50 kWh m^−2^, due, as above, to the reduction of the air temperature.

Indeed, in the CIM model the air temperature due to the built environment is reduced by 0.3°C (from 10.2°C to 9.9°C) on average per year and the wind speed is reduced by 1.5 ms^−1^ on average per year (from 1.9 to 0.4 ms −1). This difference impacts the energy demand of buildings, by increasing the energy required to heat them. It is essential to highlight that this variation is not linear throughout the year (see Table 3): the air temperature is higher in CIM during the summer time (up to +1.2°C during the month of June) and lower during the winter time (up to −2.0°C during the month of January). Additionally, the profile varies throughout the day, and, as an example, during the heating season (from October to May) the averaged air temperature is lower during the nighttime (-2.7°C) and higher during the daytime (+1.0°C). In fact, the air temperature is lower during the daytime during the coldest months (from November to February) then it starts to rise, due to the solar irradiance received and stored by the built environment. This is related to the urban environment and the sun height: effectively, during the winter time the sun is lower, consequently less radiation is received by the urban canyons. This phenomenon is refered to the so called Cold Air Pools (CAP) typical of valleys and is stronger in cloudless, windless and high-pressure conditions [33]. By comparing the CIM with the Meteonorm data, the average temperatures difference during two winter nights is higher in cloudless conditions (21st February, nebulosity equals to 2 octas) than in cloudy conditions (8th January, nebulosity equals to 8 octas), when the difference corresponds to 5°C and 2.5°C, respectively.

**Table 3:** Averaged monthly difference between the weather data provided by Meteonorm and CIM. Daytime (from 8:00 to 18:00 hours) and nighttime (from 19:00 to 7:00 hours). Adapted from [29].

Month	Wind speed(m s ^−^1)			Air Temperature (°C)		
	Daily	Daytime	Nightime	Daily	Daytime	Nightime
1	-1.6	-1.6	-1.7	-2.0	-1.2	-2.8
2	-1.9	-1.9	-1.8	-1.8	-0.1	-3.2
3	-1.8	-1.6	-2.0	-1.2	1.5	-3.5
4	-1.9	-1.9	-1.9	-0.1	3.5	-3.2
5	-1.5	-1.6	-1.5	0.7	4.7	-2.8
6	-1.6	-1.4	-1.7	1.3	5.8	-2.5
7	-1.5	-1.6	-1.4	1.2	5.5	-2.5
8	-1.2	-1.1	-1.3	0.8	4.8	-2.5
9	-1.3	-1.1	-1.4	0.6	3.6	-2.0
10	-1.3	-1.4	-1.3	-0.9	1.1	-2.5
11	-1.5	-1.5	-1.5	-1.2	-0.2	-2.1
12	-1.8	-1.6	-1.9	-1.7	-1.1	-2.3

In the current case study, the maximum difference between the models was shown for well insulated buildings, such as BC and the Rolex Learning Centre, while in the renovated one, the highest difference can be observed in the AA buildings, BC and Rolex Learning Centre, with a peak of 15% of increased demand. This difference is probably due to their form factor as well as their high façade glazing ratio (see the S1 Table for the average windows-to-wall ratio and form factor). Indeed, a non-compact building such as CH, with low glazing ratio (0.26), presents the lowest difference (9%). The fact that, due to the CIM weather profile, the energy demand of the campus increases is, somehow, not intuitive. It is clear that the reduction of the wind speed reduces the convective coefficient between the buildings and the outdoor environment, consequently reducing the heat losses, and the heating demand. But, the CIM provides also a new air temperature, based on the new wind speed, as well as on the surface temperature, as calculated by CitySim Pro. The new air temperature, in the EPFL case study, is reduced during the heating season, on average by 1.0°C from October to May.

**Fig 8:**
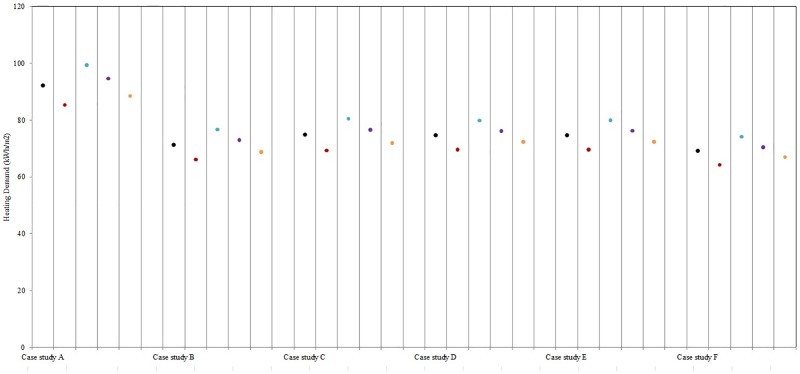
Heating demand of the five selected urban configurations (black dots), as function of the variation by +1°C (red dots) and −1°C (blue dots) of the air temperature, and by +1.5 ms^−^1 (violet dots) and −1.5 ms^−^1 (orange dots).

In order to better understand the results obtained, it is important to analyse the relative impact of varying the air temperature during the heating season. The selected configurations taken from Ratti et al., [28] are then simulated with CitySim Pro, in order to quantify their energy demand, by varying the outdoor air temperature (±1.0°C during the heating season), as well as the wind speed (±1.5ms^−^1). Fig 8 summarizes the results obtained, per each urban configuration. The parameter that mostly affects the heating demand is the variation of the air temperature: by reducing the air temperature by 1°C during the heating season, the heating demand of buildings increases (proportionally to the floor area ratio) from 7.7% in case study A (FAR = 0.036) to 7.2% in case study F (FAR = 0.20). By reducing the wind speed by 1.5 ms^−^1, the heating demand decreases by 4.0% in case study A, to 3.0% in case study F. Based on these results, it is evident that the reduction of 1°C has a higher impact in the heating demand, compared to the reduction of the wind speed. Naturally the more compact the buildings are, the less they are influenced by the environmental microclimatic variations.

**Fig 9:**
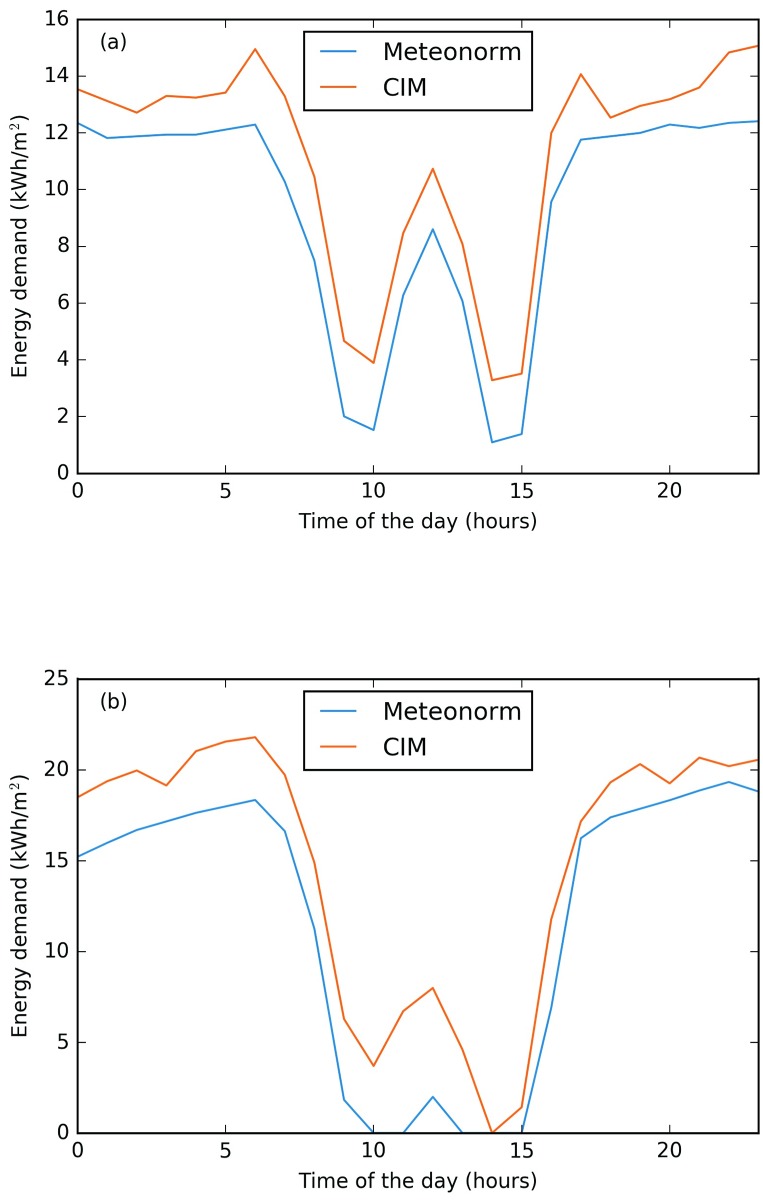
Building AAB, heating demand during (a) a winter cloudy and (b) sunny day. CIM dark line and Meteonorm light line. Inserted figure on top right represents AAB building.

**Fig 10:**
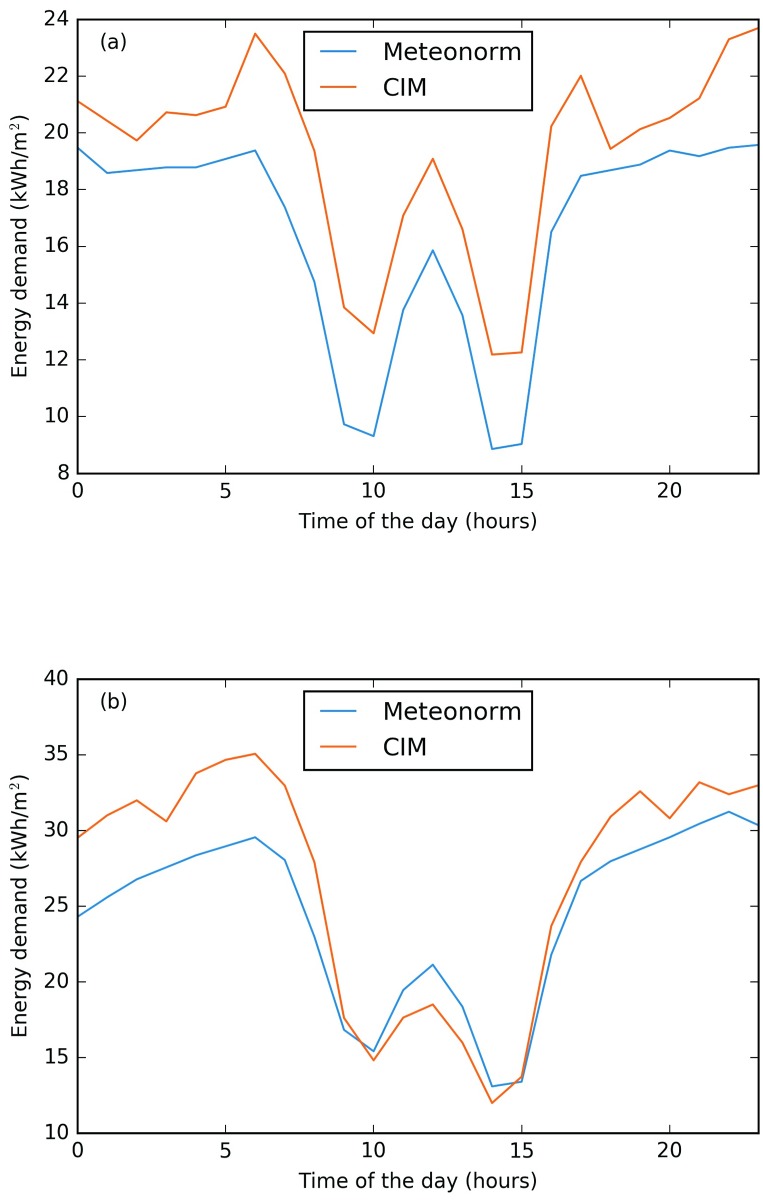
Building CH, heating demand during (a) a winter cloudy and (b) sunny day. CIM dark line and Meteonorm light line. Inserted figure on top right represents CH building.

These results also corresponds to what was calculated for the EPFL campus. Buildings with an elevated form factor and glazing ratio, such as RLC (form factor of 1.94), are more sensitive to climatic variation than compact buildings, such as BC (form factor of 0.99). This is clearly related to the surfaces exposed to the outdoor environment. In order to better understand this fact, the thermal behaviour of two buildings, AAB and CH, are analysed (more details can be found in S1 Table). The analyses are performed during two winter days: a cloudy day (22nd of January) and a sunny day (14th of February). Both Building AAB (see Fig 9) and Building CH (see Fig 10), have a higher heating demand on a cloudy day when using the CIM weather data. On the contrary during a sunny day it can be noticed that Building AAB has a higher heating demand while during most of the day for Building CH the demand is very close to the Meteonorm one and even slightly lower from 11h00 to 15h00. Indeed, Building AAB is mostly influenced by the solar gains, and its heating demand corresponds to 0 Wh m^−2^ during the hottest daytime hours. By contrast, Building CH is less influenced by the sun, and its minimal demand corresponds to 12 Wh m^−2^, but in this case the lower demand is given by the CIM weather file. This result is quite interesting, as it shows the sensitivity of this building to the wind speed and air temperature, due to its architectural design. The selected day (14th of February) is characterized by high wind speed according to the Meteonorm data set (average wind speed equals to 2.99 m s^−1^), while by contrast the same day with CIM presented a low wind speed (equals to 0.06 m s^−1^).

**Fig 11:**
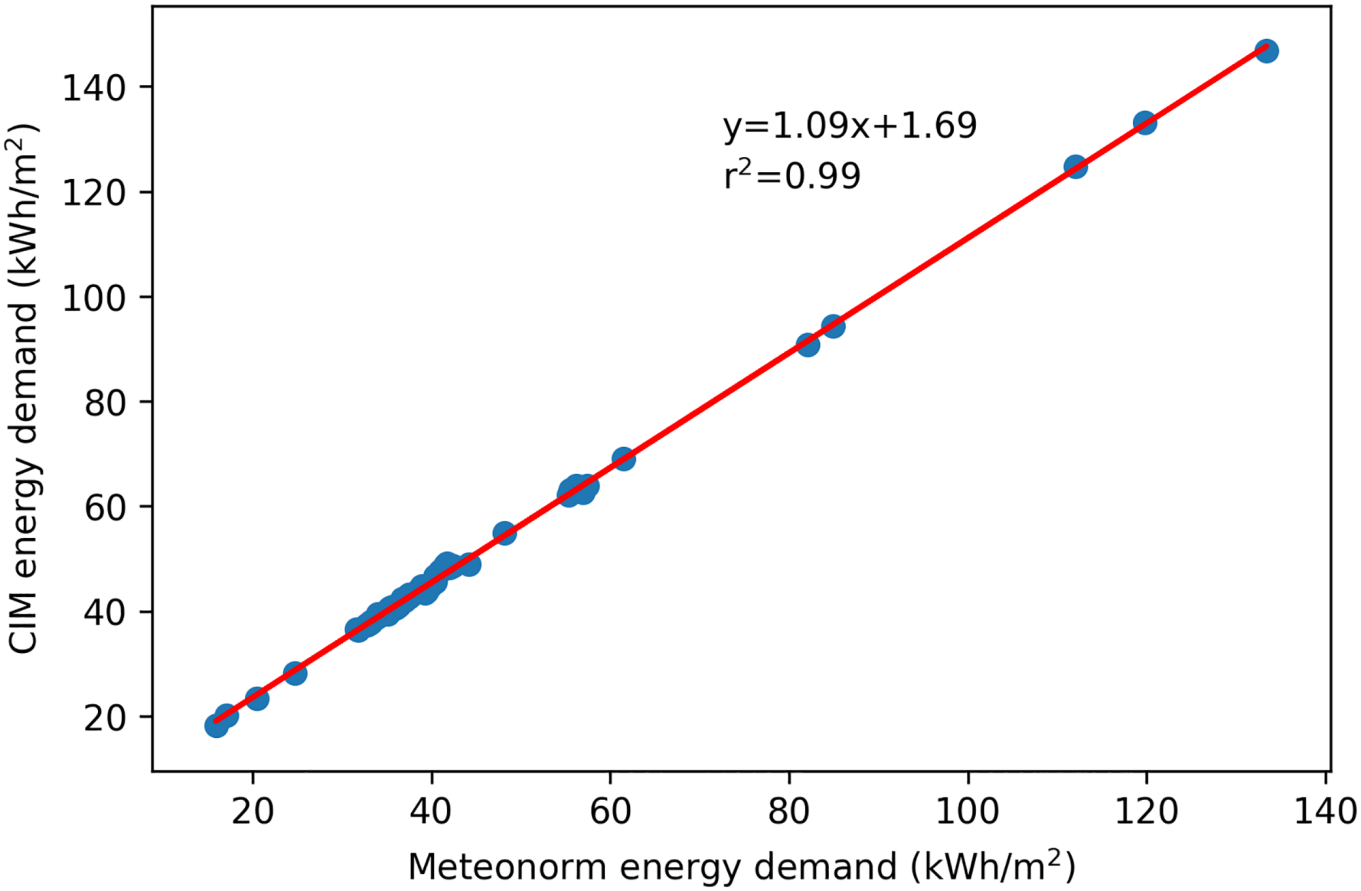
Correlation between the heating demand (kWh·m-2) calculated with CIM and Meteonorm.

The correlation between the two simulations of energy demand is clear (Fig 11) but the trend shows that when using the CIM model, the calculated energy demand for heating of the site increases. Looking at the sensitivity of each weather data on the refurbished campus according to Minergie-P, it can be seen that in both cases (Meteonorm and CIM) the demand is reduced by 44% and 43% respectively.

Finally, we confirm the capacity of CIM to simulate the actual energy consumption for the whole EPFL campus using data from the year 2015. The total energy consumption from the EPFL site for the year 2015 for heating was 30,000 MWh. If we use data for 2015 at the same height above ground as those typically given by the Meteonorm dataset (wind speed and direction at 10 m and temperature at 2 m) the heating demand for the campus was 34,433 MWh (15% difference with the actual consumption). Using the CIM data, the total heating demand simulated with CitySim was 32,582MWh (8% difference), thereby decreasing the error by a factor of 2.

## Conclusion and perspectives

The present study aimed at bridging a gap in building energy simulation by providing useful and accurate local climatic data. For this purpose, a new methodology was designed to couple a meteorological model and a building energy software. The Canopy Interface Model [14] was linked to the CitySim software [12].

It was demonstrated that CIM was able to reproduce the meteorological data collected on the EPFL campus on top of the building and close to the ground for both the wind speed and the air temperature. Although there was a very good agreement between the simulated results and collected data, the wind speed close to the ground was slightly underestimated by CIM. The main reasons for this could be that CIM overestimates the drag force and it resolves the flow in one direction and hence does not integrate all the turbulent processes.

The data from CIM were then used to simulate the heating demand for the EPFL campus for the year 2015. It was shown that there was an 8% error between the actual measured consumption and the CIM-CitySim computation.

Furthermore, data from the Meteonorm dataset, more commonly used for building energy simulation, were first used to calculate the energy consumption directly and secondly as the boundary conditions for CIM-CitySim. When taking into account the local urban environment, the variation between the two climatic scenarios leads to the following conclusions:

- The air temperature is reduced during the winter time and increased during the summer season. The variation impacts the heating demand, which is increased due to the higher thermal losses through the envelope.
- The wind speed is also reduced in the CIM weather profile. This variation increases the surface temperature of buildings, and consequently reduced the heat losses through the envelope. Some buildings are more sensitive to climatic data while other buildings are dominated by their internal gains and the climatic data only slightly impacts their energy demand. Furthermore, it was shown that the compactness of the buildings has a significant influence on the impact of the climatic data. Building with a higher form factor were found to be more sensitive to the variation of the climatic data.

Further research and work are needed to improve the complex phenomenon of the urban environment in simulations tools. For example, a two-way coupling between CitySim and CIM and could be made to create a more dynamic simulation. Besides, there is also a need to improve the parameterization of the turbulent processes in CIM without resolving 3D flows which would significantly increase the computational time. This can be achieved with the use of monitored data, such as those currently generated in the MoTUS project. High frequency monitored data are essential for the development of new parameterization of obstacle effects in urban areas.

## Supporting information

S1 Table Characteristics of Rolex Learning Centre, BC, AAB and CH buildings. Adapted from [29]. FA is the floor area, IS is the internal surface, FF is the form factor, Cons. is the period of construction and w-t-w is the averaged windows to wall ratio for the whole building.
(PDF)
