# Supplementary material for: Multi-scale modelling to evaluate building energy consumption at the neighbourhood scale
Source: PLoS One. 2017 Sep 7;12(9):e0183437. doi: 10.1371/journal.pone.0183437 (PMC5589157; doi:10.1371/journal.pone.0183437)
Supplement: S1 Table — Adapted from [29]. FA is the floor area, IS is the internal surface, FF is the form factor, Cons. is the period of construction and w-t-w is the averaged windows to wall ratio for the whole building. (PDF) [file pone.0183437.s001.pdf]

| **Building** | **Enveloppe ($m^2$)** | **FA ($m^2$)** | **IS ($m^2$)** | **FF (-)** | **Cons.** | **w-t-w** |
|---|---|---|---|---|---|---|
| Rolex | 23 035 | 15 897 | 15 970 | 1.94 | 2010 | 0.7 |
| BC | 6 956 | 2 767 | 12 796 | 0.65 | 2002 - 2004 | 0.5 |
| AAB | 3 138 | 1 048 | 3 144 | 1.16 | 1992- 2002 | 0.3 |
| CH | 18 725 | 8 382 | 25 236 | 0.91 | 1972- 1984 | 0.2 |
